# Pathophysiological role of ion channels and transporters in hepatocellular carcinoma

**DOI:** 10.1038/s41417-024-00782-8

**Published:** 2024-07-24

**Authors:** Li Zhang, Hong Gu, Xin Li, Yongfeng Wang, Shun Yao, Xingyue Chen, Liming Zheng, Xingyue Yang, Qian Du, Jiaxing An, Guorong Wen, Jiaxing Zhu, Hai Jin, Biguang Tuo

**Affiliations:** 1 Department of Gastroenterology, Digestive Disease Hospital, Affiliated Hospital of Zunyi Medical University, Zunyi, Guizhou China; 2 The Collaborative Innovation Center of Tissue Damage Repair and Regenerative Medicine of Zunyi Medical University, Zunyi, Guizhou China

**Keywords:** Targeted therapies, Targeted therapies

## Abstract

The incidence of hepatocellular carcinoma (HCC) has continued to increase annually worldwide, and HCC has become a common cause of cancer-related death. Despite great progress in understanding the molecular mechanisms underlying HCC development, the treatment of HCC remains a considerable challenge. Thus, the survival and prognosis of HCC patients remain extremely poor. In recent years, the role of ion channels in the pathogenesis of diseases has become a hot topic. In normal liver tissue, ion channels and transporters maintain water and electrolyte balance and acid‒base homeostasis. However, dysfunction of these ion channels and transporters can lead to the development and progression of HCC, and thus these ion channels and transporters are expected to become new therapeutic targets. In this review, ion channels and transporters associated with HCC are reviewed, and potential targets for new and effective therapies are proposed.

## Introduction

Hepatocellular carcinoma (HCC) originates in liver cells and is the most common primary liver cancer (90% of all liver cancer cases). HCC is the sixth most common cancer worldwide and the third most common cause of cancer-related deaths [1]. Although common serological tests can be used to diagnose HCC at an early stage, effective drugs for the treatment of hepatocellular carcinoma are still lacking [2]. Although liver transplantation is recognized as the most effective treatment for hepatocellular carcinoma, only a very small number of patients can undergo liver transplantation. Therefore, it is particularly important to identify effective therapeutic targets to better understand the etiology of HCC. In recent years, ion channels have been shown to play an important role in the pathogenesis of cancer and have become a popular research topic [3]. Ion channels and transporters are hydrophilic protein micropores in cell membranes that mediate the transcellular transport of water and ions. The high selectivity of ion transmission results in the asymmetric distribution of some ions (K^+^, Na^+^, Ca^2+^, H^+^, HCO3^−^ and Cl^−^) on both sides of the membrane, thus forming an electrochemical gradient and maintaining the physiological function of the organism. This uneven distribution of ions is necessary for the survival and function of all cells. In normal hepatocytes, ion channels and transporters primarily regulate the transport of various ions and water as well as electrolyte balance and acid‒base homeostasis, thereby maintaining the stability of the internal environment. Studies have shown that changes in ion channels and transporters are related to the pathogenesis of liver cancer. In recent years, the relationship between ion channels and tumors has gradually garnered increased attention, and continuous studies have shown that ion channels play an important role in the development of tumors. It is possible that growth factors act directly or indirectly on ion channels through signal transduction pathways to regulate the membrane potential, cell cycle, intracellular calcium ion concentration, cytoplasm, pH and cell volume, which affect the proliferation, apoptosis and differentiation of tumor cells. A thorough understanding of the role of ion channels in the occurrence and development of hepatocellular carcinoma would not only contribute to a more comprehensive understanding of the pathogenesis of cancer but may also provide new targets and novel biological interventions for the prevention and treatment of HCC.

## Hydrogen ion channels

### Sodium-hydrogen exchangers

**Fig. 1:**
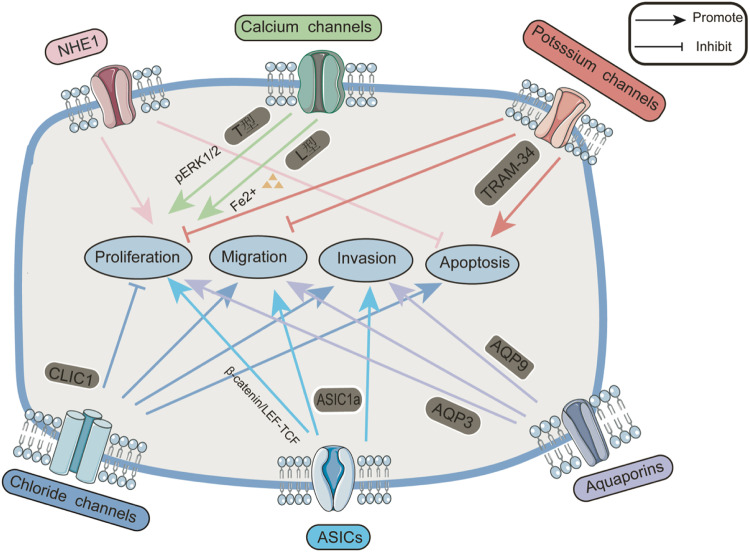
Pathological role of ion channels and transporters in hepatocellular carcinoma. Altered and dysfunctional hydrogen ion channels, calcium ion channels, potassium ion channels, chloride ion channels, acid-sensitive ion channels, and water channels have implications for proliferation, migration, invasion, and apoptosis in hepatocellular carcinoma. NHE1 Na(+)/H(+) exchanger type 1 isoform, ASICs acid-sensing ion channels, CLIC1 intracellular chloride channel 1, AQP3 aquaporin 3, AQP9 aquaporin 9, TRAM-34 triarylmethane-34, ERK extracellular signal-regulated kinase.

Na^+^/H^+^ exchange protein-1 (NHE1, also known as SLC9A1) is a universally expressed integrated membrane protein present in every cell and was the first subtype of NHE to be identified [4]. It belongs to the cation/proton antitransporter (CPA) superfamily [5] and is a secondary transporter [6] that catalyzes the exchange of intracellular proton (H^+^) ions with extracellular sodium (Na^+^) ions [7, 8]. An alkaline pH in the intracellular environment is favorable for cell growth, while an acidic extracellular environment is more likely to lead to protumor behavior. The most important function of NHE1 is to regulate the pH so that the intracellular and extracellular pH is balanced [9]. Therefore, this protein has an important role in cells that are acid‒base unbalanced under stress, and this function is also believed to exist in tumor cells. Due to the rapid proliferation of tumor cells, most of them are in an anaerobic environment and use glycolysis to generate energy [10]. Thus, they produce a high level of acidic metabolites. This acidic pH environment is not conducive to the growth of normal cells or tumor cells. The literature also suggests that an elevated intracellular pH can promote cell proliferation and inhibit apoptosis [11]. Therefore, tumor cells must initiate proton transport and maintain a relatively stable intracellular and extracellular pH by upregulating channel proteins to pump out intracellular H^+^ [12]. A more alkaline intracellular environment and more acidic extracellular environment are conducive to tumor growth and invasiveness, and this ability to upregulate proton transport channels is essential for tumor cell survival. Therefore, tumor cells often reduce intracellular H^+^ by upregulating the expression of NHE1 to transport excess H^+^ to the extracellular space [13–15]. NHE1 is a ubiquitous, amiloride-sensitive, growth factor-activable exchanger whose role is associated with cell cycle regulation, apoptosis, and tumor formation [16]. NHE1 is involved in cell-to-cell interactions in hematopoietic cells, and this cell-to-cell exchange activates NHE1, which leads to an increase in the intracellular pH and contributes to the formation of hematopoietic colonies [17]. Thus, activation of NHE1 in leukemic cells leads to a higher intracellular pH than in normal cells, and apoptosis of leukemic cells is accompanied by an increase in intracellular pH after targeted inhibition of the Na+/H+ exchanger [16]. However, H^+^ diffuses poorly in the tumor microenvironment, which results in a low extracellular pH of 6.2–6.5 [9, 18]. Thus, the presence of NHE1 promotes the proliferation of tumor cells. Studies have demonstrated that NHE1 plays an important role in tumor growth and differentiation [19]. In addition, NHE1 is critical in the proliferation and migration of tumor cells [20–23] (Fig. 1). The above experimental findings indicate that NHE1 is involved in tumor proliferation, differentiation, and migration. Some studies have shown that in cancers such as liver hepatocellular carcinoma (LIHC), the expression of NHE1 in tumor tissues is significantly greater than that in normal tissues, and that the higher the expression of NHE1, the higher the degree of malignancy of LIHC [19]. Yang et al. [24] reported that NHE1 expression is increased in HCC cells and tissues relative to normal hepatocytes and tissues, and its increased expression is positively correlated with tumor size, venous infiltration, and tumor stage. In contrast, in one study, when NHE1 expression was inhibited via NHE1 and EIPA knockdown ([5-(N-ethyl-N-isopropyl)], a highly specific NHE1 inhibitor), HCC cell growth was suppressed to a certain extent, and apoptosis was increased. Therefore, NHE1 could be an important biomarker for the prognosis of hepatocellular carcinoma patients and a new target for HCC immunotherapy. Animal models have shown that EIPA inhibits the growth of mouse hepatocellular carcinoma that formed after transplantation of tumor cells. These data suggest that NHE1 may play a role in the growth and progression of HCC. Both of these experimental studies confirmed the important role of NHE1 in HCC. However, the mechanisms underlying the specific roles of NHE1 in HCC have not been elucidated and require further exploration in experimental studies. However, NHE1 is likely a potential therapeutic target for HCC.

### Calcium channels

Calcium channels are a class of transmembrane glycoproteins that are widely present in all tissue types and regulate the transport of calcium ions. Some calcium channels are voltage-dependent (L-type, T-type, and N-type), while others are receptor-manipulated channels. The more well studied channels are voltage-dependent calcium channels. Voltage-gated calcium channels are multisubunit complexes consisting of a central pore-forming subunit and multiple auxiliary subunits [25]. Voltage-gated calcium channels present in the cell membrane are activated by depolarization, after which calcium ions enter the cell to initiate many physiological responses, such as secretion, muscle contraction and gene transcription [25, 26]. Calcium ions participate in many signaling pathways as second messengers and play a major role in the cell cycle, cell proliferation, differentiation and apoptosis [27]. Intracellular Ca^2+^ release and extracellular Ca^2+^ entry are the most fundamental signaling pathways of the cell cycle and cell proliferation. Under normal conditions, cyclic adenosine monophosphate (cAMP) inhibits the growth of cells, and disruption of Ca^2+^ mobilization transforms these cells into a growth phenotype stimulated by cAMP [28]. Thus, intracellular Ca^2+^ homeostasis is associated with normal cell growth. In addition to these basic roles of Ca^2+^ in organisms, inhibitors of glycolysis and oxidative phosphorylation have been shown to cause an increase in intracellular Ca^2+^ in perfused rat livers [29]. Calcium channel blockers have also been reported to exert a protective effect against hepatocellular injury, especially against that caused by various toxic drugs [30]. Of the plasma membrane Ca^2+^-ATPases (PMCAs 1–4), isoforms 1 and 4 are ubiquitous, and 2 and 3 are present in only a few cell and tissue types; their main function is to transport Ca^2+^ from the cytoplasm to the extracellular compartment, thereby maintaining intra- and extracellular Ca^2+^ homeostasis [31]. In a study of liver PMCA subtypes, differences in PMCA activity were associated with differential expression of mRNAs encoding different subtypes of PMCAs. mRNAs of different PMCA isoforms are differentially expressed during normal liver development and in adulthood, as well as during regeneration after hepatectomy and in hepatocellular carcinoma cells [32]. This implies that PMCAs function in fine-tuning Ca^2+^ regulation in important cellular processes in the liver that are directly related to calcium homeostasis.

SERCA is a transmembrane pump encoded by three genes (ATP2A1-3), which allow for multiple isoforms and splice variants of SERCA (SERCA1a-b, SERCA2a-d, and SERCA3a-f) [33]; this pump plays an important role in muscle contraction primarily via the translocation of Ca^2+^ from the cytoplasm to the sarcoplasmic reticulum (SR) in skeletal and cardiac myocytes as well as in the endoplasmic reticulum (ER) in nonmuscle cells [34]. ER stress is a protective stress response of cells that can lead to apoptosis or death, and SERCA is a potential target protein that participates in ER stress. In the study by Zhang et al., palmitic acid induced ER stress and significantly inhibited the activity of SERCAs, which led to calcium overload and severe liver damage in the cytoplasm of nontransfected HSS (hepatic stimulator substance) cells, whereas in HSS -transfected cells, calcium overload was significantly overcome by the increase in SERCA activity, and liver damage was reduced [35]. This shows that the regulation of Ca^2+^ by SERCA functions in protecting the liver.

Thus, the physiological role of Ca^2+^ in the liver should not be underestimated. Some relevant studies have shown that T-type calcium channels are associated with cancer cell proliferation [36, 37]. Li et al. [38] reported that blocking functional T-type Ca^2+^ channels significantly reduced the proliferation of HCC cells. Then, they investigated genes related to cell proliferation and the cell cycle and analyzed their possible roles in signal transduction pathways in tumor cells. An in-depth study revealed that pERK1/2 (phosphorylated extracellular signal-regulated kinase, ERK) may be involved in this process. Currently, no specific mechanism by which T-type calcium channels promote HCC proliferation has been defined; thus, only the potential role of pERK1/2 provides a new research direction. Subsequent in-depth studies of this trial will aid in the selection of better treatment strategies for liver cancer patients. Another study on Ca^2+^ and hepatocellular carcinoma reported that the delivery of amplitude-modulated 27–12 MHz radiofrequency electromagnetic fields at tumor-specific frequencies (AM RF EMF) showed anticancer activity both in vitro and in patients with advanced HCC [39, 40]. Jimenez et al. [41] showed that hepatocellular carcinoma cells treated with AM RF EMF exhibited shrinkage, which was due to the differentiation of tumor cells from their original morphology to quiescent cells with a spindle morphology, leading to shrinkage. Upon further investigation, it was found that under AM RF EMF conditions, the inward flow of calcium through the Cav3.2 T-type voltage-gated calcium channel (CACNA1H) was initiated [41]. This led to an increase in the intracellular calcium concentration in HCC cells, which resulted in the targeted inhibition of HCC cell growth and downregulation of cancer stem cells. This is a tumor- and tissue-specific process, which suggests a role for intracellular downstream effects induced by the inward flow of cytoplasmic Ca2+ [42]. In conclusion, their experimental study demonstrated the significance of tumor-specific amplitude-modulated radiofrequency electromagnetic fields in inducing the differentiation of hepatocellular carcinoma cells by targeting CACNA1H and Ca^2+^ influx. This provides a new direction for research on a disease that is always treated with conventional therapies and rarely has treatment breakthroughs. The importance of this study is substantial, as CACNA1H may have a role in the diagnosis and treatment of various forms of cancer, including liver cancer. Moreover, this study provides ideas for therapeutic research on other cancers. Studies have shown that L-type calcium channels play a role in iron uptake in hepatocellular carcinoma cells, which ultimately promotes the proliferation of hepatocellular carcinoma cells. However, the exact mechanism is not clear [43]. Therefore, the use of calcium channel blockers to inhibit iron uptake in hepatocellular carcinoma cells has the potential to be an alternative approach for the treatment of HCC, but further studies are needed to explore this treatment option. Existing studies have shown that voltage-gated calcium channels are associated with the development of hepatocellular carcinoma, and to a large extent, they have provided new directions for the treatment of this cancer. However, the specific underlying mechanism has not been elucidated and still requires further exploration in multiple experimental studies to identify specific targets for the treatment of hepatocellular carcinoma.

### Potassium channels

Potassium ion channels are pore-forming transmembrane proteins that are broadly categorized into four types: (1) voltage-gated channels; (2) calcium-dependent channels; (3) inwardly rectifying channels; and (4) two-pore domain channels [44, 45]. Potassium channels perform normal physiological functions by mediating intercellular electrical signaling and maintaining cellular ion homeostasis. Potassium channels affect the cell cycle, proliferation, migration and apoptosis of a wide range of normal and cancer cells by regulating membrane potential or altering intracellular signaling [46–48]. Tumor-associated potassium channels have been identified as calcium-activated, voltage-dependent, inwardly rectifying, ATP-type, and swelling-activated potassium channels [49]. Intermediate conductance Ca-activated K+ channel-1 (IKCa1) is highly expressed in human hepatocellular carcinoma tissues, and its specific blocker, TRAM-34 (triarylmethane-34), is able to effectively inhibit the proliferation of the human hepatocellular carcinoma cell line HepG2 [50]. Thus, IKCa1 blockers may be effective drugs for the treatment of HCC. In another study, Liu et al. [51] reported that blockade of the conductance calcium-activated potassium channel (KCa3.1) by TRAM-34 inhibited the proliferation of HepG2 cells and induced apoptosis. Additionally, TRAM-34 significantly inhibited the migration of HepG2 cells but increased their apoptosis rate. Moreover, cell proliferation and migration were similarly inhibited after KCa3.1 channel knockdown. Further studies showed that blocking KCa3.1 inhibited the growth and migration of human hepatocellular carcinoma cells and promoted apoptosis by regulating intracellular ROS levels and promoting p53 activation. Thus, intracellular ROS levels and p53 can be modulated, thereby achieving the goal of treating HCC with potassium channel blockers. Previous studies have shown that potassium channel blockers can inhibit the proliferation and migration of HCC cells while promoting apoptosis. Therefore, the potassium channel blocker TRAM-34 is a promising anti-hepatocellular carcinoma drug, but further studies are needed for confirmation. In recent years, the Na^+^/K^+^-ATPase alpha subunit has emerged as a new target for anticancer therapy [52]. Na^+^/K+-ATPase is closely related to cancer development, progression and migration, in addition to its key role in ion transport, metabolism and signal transduction [53]. Xu et al. [54] showed that the Na^+^/K^+^-ATPase alpha 1 subunit was highly expressed in HCC cells and that HCC cell proliferation was inhibited after Na^+^/K^+^-ATPase activity was inhibited by ouabain (a natural inhibitor of Na^+^/K^+^-ATPase) or siRNA blockade. The possible mechanism is that ouabain decreases Na^+^-K^+^ exchange and increases the intracellular Na+ concentration by inhibiting Na^+^-K^+^-ATPase activity in HCC cells [55]. This causes depolarization of the cell membrane, which activates voltage-dependent Ca^2+^ channels and leads to an increase in the inward flow of extracellular Ca2+. In addition, inhibition of Na^+^/K^+^-ATPase activity by ouabain results in a decrease in the level of ATP dephosphorylation, which promotes the phosphorylation of Na^+^/K^+^-ATPase and increases the permeability of the cellular membrane so that extracellular Ca^2+^-induced release of Ca^2+^-bound calcium-binding proteins (CABPs) can occur [56]. All these effects may lead to an increase in the intracellular Ca^2+^ concentration, and intracellular Ca^2+^ overload is an important cause of apoptosis and necrosis [57]. In addition, ouabain reduces glycolysis levels in HCC cells and decreases the production of nicotinamide-adenine dinucleotide phosphate (NADPH), which is necessary for the clearance of ROS (Reactive Oxygen Species) from tumor cells [54]. Elevated intracellular ROS concentrations can cause DNA damage and induce apoptosis [58]. Therefore, ouabain, an inhibitor of Na^+^/K^+^-ATPase, inhibits proliferation and induces apoptosis of HCC cells. Hepatocellular carcinoma treatment can be initiated with the Na^+^/K^+^-ATPase inhibitor ouabain, and the use of Na^+^/K^+^-ATP inhibition can also be explored as a way to target HCC. Another type of potassium channel, the voltage-gated K^+^ channel, also plays a role in the proliferation and migration of hepatocellular carcinoma cells, and HCC cell proliferation and migration are inhibited by different potassium blocking agents. This may be because the inhibition of potassium channels also reduces the inward flow of calcium ions, which affects the function of voltage-gated K^+^ channels in the adhesion and proliferation of tumor cells [59]. These studies demonstrated that potassium ion channels are involved in proliferation, migration, apoptosis, and other tumor biological behaviors in hepatocellular carcinoma and that inhibitors of potassium ions are potential therapeutic targets in HCC.

### Chloride channels

Members of four classes of chloride channels [ClC family, CLCA family, CLIC family, and mitochondrial voltage-dependent anion channels (VDACs)] are expressed in hepatocytes [60]. Chloride channels perform a variety of physiological functions related to the control of membrane potential stability, electrical excitability, intracellular ATP hydrolysis, cell volume regulation, electrolyte stabilization, and intracellular pH stabilization in all cells, including hepatocytes [61]. Chlorine channels play an important role in hepatocyte volume regulation and organelle acidification within hepatocytes [60, 62]. In contrast, hepatocyte volume regulation depends on an increase in chloride channels [63]. In addition, chloride channels function in regulating hepatocyte apoptosis and growth [60]. The most studied protein, chloride intracellular channel 1 (CLIC1), is highly expressed in human hepatocellular carcinoma and is closely associated with tumor size, distant metastasis, pathological stage, and low survival rate [64]. Another study also showed that CLIC1 is highly expressed in hepatocellular carcinoma, inhibits cell proliferation, and promotes the migration, invasiveness and apoptosis of hepatocellular carcinoma cells [65]. CLIC1 regulates cellular matrix adhesion and membrane protrusion formation by recruiting PIP5K1A/C to the cell membrane [66]. Membrane protrusion and adhesion to the extracellular matrix are essential processes for cell migration, tumor invasion, and metastasis. Studies have demonstrated that CLIC1 promotes hepatocellular carcinoma cell invasion [67]. These findings show that CLIC1 plays an important role in the development of hepatocellular carcinoma. Another type of chloride channel, the calcium-activated chloride channel (CaCC), functions in increasing the intracellular calcium ion concentration. These ions activate chloride channels, which are commonly found in a variety of cells. A mechanistic study of cyclosporine A (CsA) in hepatocellular carcinoma revealed that cyclosporine A induced apoptosis of hepatocellular carcinoma cells [68]. This process may be mediated by calcium-activated chloride channels and may result in disturbances in intracellular calcium signaling.

### Acid-sensitive ion channels

Acid-sensitive ion channels (ASICs) are H^+^-gated cation channels that are activated in response to changes in extracellular pH and are widely expressed in the mammalian central and peripheral nervous systems [69, 70]. These channels play a role in perceived pain, learned memory, and fear conditioning [71]. To date, at least six ASIC subunits (1a, 1b, 2a, 2b, 3, and 4) have been identified [72]. Of these, ASIC1a has become a popular research topic due to its important biological functions and pathological significance. Studies have shown that transporter proteins and pumps contribute to H+ secretion and that ASICs are involved in this process [73]. The expression of ASIC family members is needed for normal smooth muscle cell migration [74], and it has also been demonstrated that ASIC1a is associated with tumor cell proliferation and migration [75]. ASIC1a is highly expressed in HCC and is promoted by an acidic extracellular environment. The upregulation of ASIC1a was shown to be positively associated with advanced tumor metastasis in HCC patients, while knockdown of ASIC1a inhibits HCC migration and invasion [76]. This finding regarding HCC metastasis is important as it suggests a new anticancer gene therapy approach. However, the specific mechanism by which ASIC1a affects HCC metastasis remains unclear, and more studies are needed for further exploration. In a study by Jin et al. [77], the mRNA and protein expression levels of ASIC1a were higher in liver cancer tissues than in paracancerous tissues, and ASIC1a promoted HCC proliferation in a pH-dependent manner. Moreover, high levels of ASIC1a expression were found to be positively correlated with advanced clinical stage and poor prognosis in cancer patients. The inhibition of hepatocellular carcinoma cell proliferation after the knockdown of ASIC1a yielded the same results in nude mouse experiments. The authors also found that ASIC1a may promote cancer cell proliferation through β-catenin and nuclear accumulation, i.e., ASIC1a may act through β-catenin/LEF-TCF signaling. Knockdown of ASIC1a inhibits cancer cell proliferation and tumorigenicity in vitro and in vivo through β-linker protein degradation and LEF-TCF inactivation. This finding also suggests that ASIC1a is a potential diagnostic marker and chemotherapeutic target in hepatocellular carcinoma, which provides a new direction for the treatment of HCC.

### Aquaporins

Aquaporins (AQPs) are membrane channel proteins that promote the rapid passive transport of water and facilitate the transmembrane transport of glycerol and urea. Under special conditions, AQPs can also promote the transmembrane transport of CO2, ammonia and nitrate [78]. In addition to the transport of small molecules, AQPs are involved in a variety of biological processes, such as tissue swelling [79], glycolipid metabolism [80], neural signaling [81], cell migration [82] and many other processes.

Thirteen AQPs (AQP0–AQP12) have been cloned from mammals. These AQPs are categorized into (1) classical AQPs, which are essentially water permeable (AQP0, AQP1, AQP2, AQP4, AQP5, AQP6, and AQP8); (2) aquaglyceroporins, which are water permeable but are also permeable to glycerol, urea, and small solutes (AQP3, AQP7, AQP9, and AQP10); and (3) heterodimorphic AQPs (AQP11 and AQP12), which have a highly variable NPA motif [83–85]. AQPs are widely expressed in the hepatobiliary system [78] (Table 1), as hepatocytes express AQP0, AQP8, AQP9 and AQP11 [84, 86, 87]. Cholangiocytes express AQP0, AQP1, AQP4, AQP5, AQP8, AQP9 and AQP11 [88], and gallbladder epithelial cells express AQP1 and AQP8 [89, 90]. AQP1 is highly expressed in peribiliary and gallbladder vessels, which suggests that AQP1 may facilitate the movement of water from plasma to bile [88, 89]. AQP8 is associated with the permeability of the tubular plasma membrane and may play an important role in bile duct formation [78]. AQP9 is a liver-specific glycerol channel expressed on the basolateral plasma membrane of hepatocytes [91]. AQP9 is highly permeable and transports urea and many other solutes produced in hepatocytes in and out of the cell [91, 92]. Along with AQP8, AQP9 mediates water transport between the blood sinusoids and the interior of the hepatocyte and thus contributes to biliary bile secretion [86]. Studies have demonstrated that the expression levels of AQP9 are greatly increased in the livers of rats in a starvation state, which suggests that during starvation, the liver takes up glycerol for gluconeogenesis via AQP9 [92]. It has also been reported that AQP8 and 9 expression levels are significantly lower in hepatocellular carcinoma cells than in normal liver cells and that plasma membrane permeability is reduced in HCC. This leads to increased resistance of hepatocellular carcinoma cells to apoptosis [93]. Studies have shown that AQPs are frequently dysregulated in cancer and play an important role in tumor development [94]. Dysregulated expression of AQPs promotes esophageal squamous cell carcinoma [95], thyroid cancer [96], lung cancer [97] and many other malignant tumors. Compared with those in nontumor tissues in patients with HCC, the protein levels of AQP3 and AQP5 are increased in HCC tissues. In addition, the coexpression of the AQP3 and AQP5 proteins is positively correlated with elevated serum AFP levels, tumor stage, and tumor grade [98]. Thus, AQP3 and AQP5 are involved in tumor progression in HCC patients and are closely related to the prognosis of these patients. Data shows that AQP3 expression is upregulated in HCC and that high expression of AQP3 is significantly positively correlated with tumor grade, tumor stage, and lymph node metastasis in HCC patients. AQP7 and AQP9 expression levels are downregulated in HCC, and low expression levels of AQP7 are significantly positively correlated with tumor grade [99]. Although AQPs play an important role in HCC progression, further experimental studies are needed to determine the specific biological functions of specific AQPs in HCC. Chen et al. reported that miR-124 expression is downregulated in HCC tissues and that miR-124 expression inhibits the proliferation and migration of HCC cells. However, the expression of aquaporin 3 (AQP3), a direct target of miR-124, was shown to be upregulated in HCC tissues and negatively correlated with miR-124 expression. In that study, AQP3 overexpression promoted cell proliferation and migration, while miR-124 knockdown inhibited cell proliferation and migration. These studies revealed the mechanism by which circular RNA HIPK3 (circHIPK3) acts as a miR-124 sponge and regulates the expression of the miR-124 target gene AQP3 [100]. Therefore, inhibition of cell proliferation and migration by silencing the circular RNA HIPK3 (circHIPK3), which downregulates AQP3 expression, is also an effective therapeutic approach. It has been reported in the literature that the migration ability of hepatocellular carcinoma HepG2 cells is related to AQP9 expression, and the higher the AQP9 expression, the more invasive the hepatocellular carcinoma cells. The mechanism by which AQP9 promotes the migration of hepatocellular carcinoma cells is currently unclear. It is possible that AQP9 promotes the rapid transmembrane flow of water molecules, which would cause the osmotic pressure inside and outside the cell membrane to change rapidly [101]. This rapid change in osmotic pressure could promote the formation of cell plate pseudopods, thus facilitating cell deformation and migration.

**Table 1:** Expression, localization, and physiological significance of AQPs in the hepatobiliary system.

Expression	Localization	Physiological significance
AQP0	Liver lens fibers [89]	Low-volume water channel, cell‒cell adhesion molecule [89]
AQP1	Cholangiocyte apical, basolateral plasma membrane structural domains, intracellular vesicular compartments [78]; gallbladder epithelial cells [89, 90]；biliary conduit vascular plexus and gallbladder vasculature [88, 89]	Top transportation of water [102–104]
AQP3	Fat cells [105]	Glycerol channel, involved in glycolipid metabolism [105–107]
AQP4	Structural domains of the basolateral plasma membrane of cholangiocytes [104]	Modulation of basolateral motion [102–104]
AQP7	Fat cells [105]	Glycerol channels, involved in glycolipid metabolism [105–107]
AQP8	Rat: intracellular cupular plasma membrane in hepatocytes [86]; Mouse: hepatocyte endothelium [108] gallbladder epithelial cell [89, 90]	Related to tubular permeability [109]; Involved in mitochondrial volume control [110, 111]
AQP9	Hepatocyte basolateral plasma membrane [91, 112]	Involved in bile secretion from bile ducts [86]

Increasing evidence suggests that AQPs may be involved in physiological processes, such as hepatic intravascular and ductal bile secretion and gluconeogenesis, and that they may also affect other functions of the liver, which needs to be further studied and explored. Given the recently discovered role of AQPs in cell migration, hepatic AQPs may have specific functions in intracellular organelles and may be involved in liver regeneration and tumor metastasis. In this review, only six ion channels and transporters strongly related to HCC progression were identified. Other ion channels and transporters are also involved in HCC progression, but they are not described here because they are either not strongly related to HCC progression or their mechanisms are unclear. This paper only summarizes 7 ion channels and transporters that are strongly related to the progression of hepatocellular carcinoma. There are still other ion channels and transporters that are also involved in the progression of hepatocellular carcinoma, which have not been mentioned in this paper due to their weak correlation with the progression of hepatocellular carcinoma or unclear mechanism.

## Conclusion

Increasing numbers of studies have demonstrated the role of ion channels in tumorigenesis and development. Thus, the same ion channels and transporters play important roles in HCC progression. They regulate not only the tumor microenvironment but also other aspects of the cell, including drug resistance. Therefore, this review summarizes the different physiological and pathological roles of various ion channels and transporters, including acid‒base transporters, Cl^-^channels, K^+^channels, Ca^2+^channels, acid-sensitive ion channels, and AQPs, in the occurrence and development of HCC. Moreover, blocking different ion channels has also been found to inhibit HCC proliferation, metastasis, and other tumor-related processes. Thus, the use of ion channels as therapeutic targets to explore novel targeted therapies for HCC is very meaningful, but these targets still require confirmation in future research. This review provides a basic and systematic summary of the field and a new direction for the diagnosis and treatment of HCC.
